# Navigating the Complexities of Anterior Cord Syndrome in a 71-Year-Old: A Case Study of Vertebral Osteomyelitis and Methicillin-Resistant Staphylococcus aureus Bacteremia

**DOI:** 10.7759/cureus.75744

**Published:** 2024-12-15

**Authors:** Krishna Patel, Vijay Sinha, Hemaswini Kakarla, Stephen G Fletcher, Gagandeep S Grewal

**Affiliations:** 1 Internal Medicine, Merit Health Wesley, Hattiesburg, USA

**Keywords:** anterior cord syndrome, end stage renal disease (esrd), methicillin-resistant staphylococcus aureus, mri imaging, vertebral osteomyelitis

## Abstract

Anterior cord syndrome is a rare yet critical neurological condition that poses significant challenges in clinical management. We present the case of a 71-year-old male with a medical history of hypertension, uncontrolled type II diabetes mellitus, hypothyroidism, and end-stage renal disease requiring dialysis who presented to the emergency department with complaints of chills, back pain, abdominal pain, and vomiting episodes. Based on the severity of the patient's illness, it was decided that inpatient admission would be best. At the beginning of the patient’s hospital stay, blood cultures grew methicillin-resistant Staphylococcus aureus. In addition, the results of the MRI revealed progressive vertebral body osteomyelitis and discitis at the T10-T11 level, along with surrounding inflammation and edema. During the patient’s hospital stay, the patient’s neurological condition worsened, and he presented with the onset of bilateral lower limb flaccid paralysis, numbness, and weakness. An MRI of the thoracic spine with and without contrast revealed worsening osteomyelitis and discitis at T10-T11. Given these findings, neurology and orthopedic surgery were consulted. The neurology team diagnosed the patient with anterior cord syndrome. The orthopedic surgery team recommended decompression and a posterior laminectomy. Due to the patient’s pre-existing pulmonary issues, uncontrolled type II diabetes mellitus, and chronic kidney disease, the orthopedic surgery team decided to proceed with a posterior decompression through the transpedicular route. Given the presence of bacteremia, a Hickman catheter was placed in the right subclavian vein for continued IV antibiotic treatment. On the last day of hospitalization, the patient was discharged to a long-term acute care facility for continued treatment. Collaboration amongst hospitalists, infectious disease specialists, neurosurgeons, and rehabilitation teams is required in order to facilitate early recognition of critical clinical symptoms in managing complex MRSA-induced spinal cord infections. The complexity of this case is due to the patient’s comorbid conditions. Correlating the symptoms of constipation and bladder dysfunction to a neurogenic cause was a challenge for this patient in the setting of hypothyroidism and opioid usage. In summary, early detection of infection and promptly working with physical therapy are critical in order to prevent catastrophic neurological consequences in patients with multiple comorbidities.

## Introduction

Anterior cord syndrome is characterized by loss of pain and temperature and motor paralysis/dysfunction due to damage to the lateral spinothalamic tract and corticospinal tract. However, modalities carried by the dorsal column, such as proprioception, vibration, and two-point discrimination, are spared [1]. In the literature, anterior cord syndrome is described as the most common type of spinal cord infarction [1]. The etiology of anterior cord syndrome includes direct anterior cord compression, which can come from pathologies such as disc protrusion, posterior abdominal aortic aneurysm, or any mass effect on the spinal cord, including infectious causes that may result in epidural abscess (e.g., Staphylococcus aureus), spinal cord abscess, Pott's disease (tuberculosis), parasitic infections (e.g., schistosomiasis, neurocysticercosis, hydatid disease), fungal infections (e.g., Cryptococcus neoformans, Aspergillus, Coccidioides), granulomatous diseases (e.g., tuberculomas, sarcoidosis), viral infections (e.g., herpes or varicella-zoster), and brucellosis [1]. Anterior cord syndrome can also result from a hyperflexion injury of the cervical spine or thrombosis of the anterior spinal artery (ASA) [1]. In the literature, infectious causes of spinal cord infarction are considered rare compared to vascular disorders or trauma [1].

Vertebral osteomyelitis can lead to compression of the spinal cord or cauda equina due to abscesses or bone softening and can also spread to adjacent tissues, nerve roots, and the epidural space, causing inflammation and abscesses of the spinal cord, possibly leading to spinal cord infarction resulting in anterior cord syndrome. Systemic antibiotic therapy is currently the main treatment for vertebral osteomyelitis [2]. After treatment, serial scans and imaging studies are necessary to ensure healing is occurring [2]. Physical rehabilitation is also recommended to restore muscle strength in cases where neurologic function declines [2]. We report the case of a 71-year-old male known to have hypertension, uncontrolled type II diabetes mellitus, and end-stage renal disease dependent on dialysis, who developed vertebral osteomyelitis from methicillin-resistant Staphylococcus aureus (MRSA) bacteremia from dialysis catheter manipulation resulting in anterior cord syndrome.

## Case presentation

A 71-year-old male, height 182.88 cm, weight 108.86 cm, with a medical history of hypertension, uncontrolled type II diabetes mellitus, hypothyroidism, and end-stage renal disease (ESRD) requiring dialysis, presented to the emergency department (ED) with complaints of chills, back and abdominal pain, and episodes of vomiting. The patient noted that his back and abdominal pain began approximately five weeks after his dialysis port was moved from the right internal jugular vein to the left internal jugular vein at an outpatient hospital.

A physical examination was conducted where he exhibited pain radiating to his back and tenderness in the right lower quadrant. Vital signs on admission were notable for a blood pressure of 178/84 mmHg, a heart rate of 115 beats per minute (bpm), and an oxygen saturation of 94% on room air.

**Figure 1:**
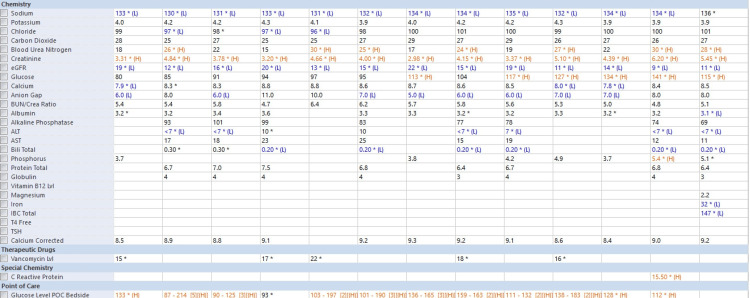
Additional laboratory analysis on admission Laboratory results highlight altered values on a comprehensive metabolic panel with abnormalities indicative of inflammation or infection including an abnormal iron panel, consistent with the patient’s medical history and clinical presentation. eGFR: estimated glomerular filtration rate; ALT: alanine aminotransferase; AST: aspartate aminotransferase; BUN: blood urea nitrogen; TSH: thyroid stimulating hormone

Initial laboratory investigations (Table 1) revealed elevated inflammatory markers and mild anemia. Additional tests (Figure 1) confirmed hypothyroidism, and imaging showed signs of potential spinal infection. As part of the primary team's decision to treat the patient promptly, a contrast-enhanced CT scan of the abdomen and pelvis was initially performed to screen for possible spinal infection and showed lytic alterations at T10-T11 and perivertebral soft tissue density, raising concerns about discitis and osteomyelitis (Figure 1). Given these findings, the neurosurgery team was consulted and recommended an MRI of the thoracic spine without intravenous contrast for further evaluation.

**Table 1:** Laboratory Investigations on admission Laboratory values highlight elevated white blood cell count, indicative of inflammation or infection, and abnormalities in renal and thyroid function, consistent with the patient’s medical history and clinical presentation.

Parameter	Result	Reference Range	Interpretation
White Blood Cell Count	11.3 µL	4.5-11.0 µL	Increased
Hemoglobin	9.6 g/dL	13.5-17.5 g/dL (male)	Decreased
Blood Urea Nitrogen	29 mg/dL	7-20 mg/dL	Increased
Creatinine	5.87 mg/dL	0.6-1.3 mg/dL	Increased
Total T4	3.8 µg/dL	4.5-12.0 µg/dL	Decreased
Thyroid-Stimulating Hormone	9.800 mIU/mL	0.4-4.0 mIU/mL	Increased

**Figure 2:**
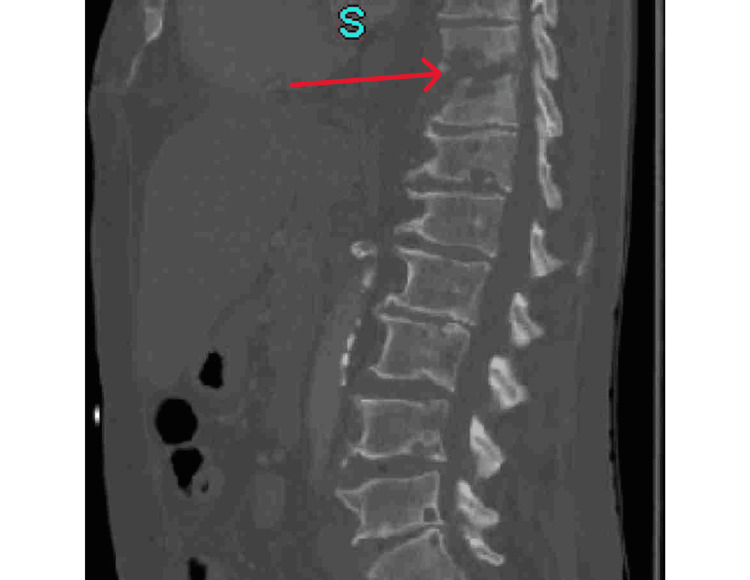
Contrast-enhanced CT scan of the abdomen and pelvis Extensive lytic changes involving the T10 and T11 vertebra, centered around the disc space, with perivertebral soft tissue density. Findings concerning for osteomyelitis/discitis.

Between day three and day eight of the patient’s hospital stay, blood cultures grew methicillin-resistant Staphylococcus aureus (MRSA). In addition, the results of the MRI revealed progressive vertebral body osteomyelitis and discitis at the T10-T11 level, along with surrounding inflammation and edema (Figure 2). In light of the absence of an epidural abscess, the neurosurgery team recommended a conservative treatment regimen that included bracing and intravenous (IV) antibiotics. After consulting with the infectious disease team, IV vancomycin was started to combat MRSA. The nephrology team was then consulted because of the patient’s pre-existing ESRD. The nephrology team suggested that the patient's ESRD be treated with dialysis and that his blood pressure, renal osteodystrophy, and anemia be closely monitored through the use of daily complete blood counts and basic metabolic panels.

Between days nine and 19 of the patient’s hospital stay, the left internal jugular hemodialysis catheter was entirely extracted due to the patient’s protracted invasive MRSA infection.

**Figure 3:**
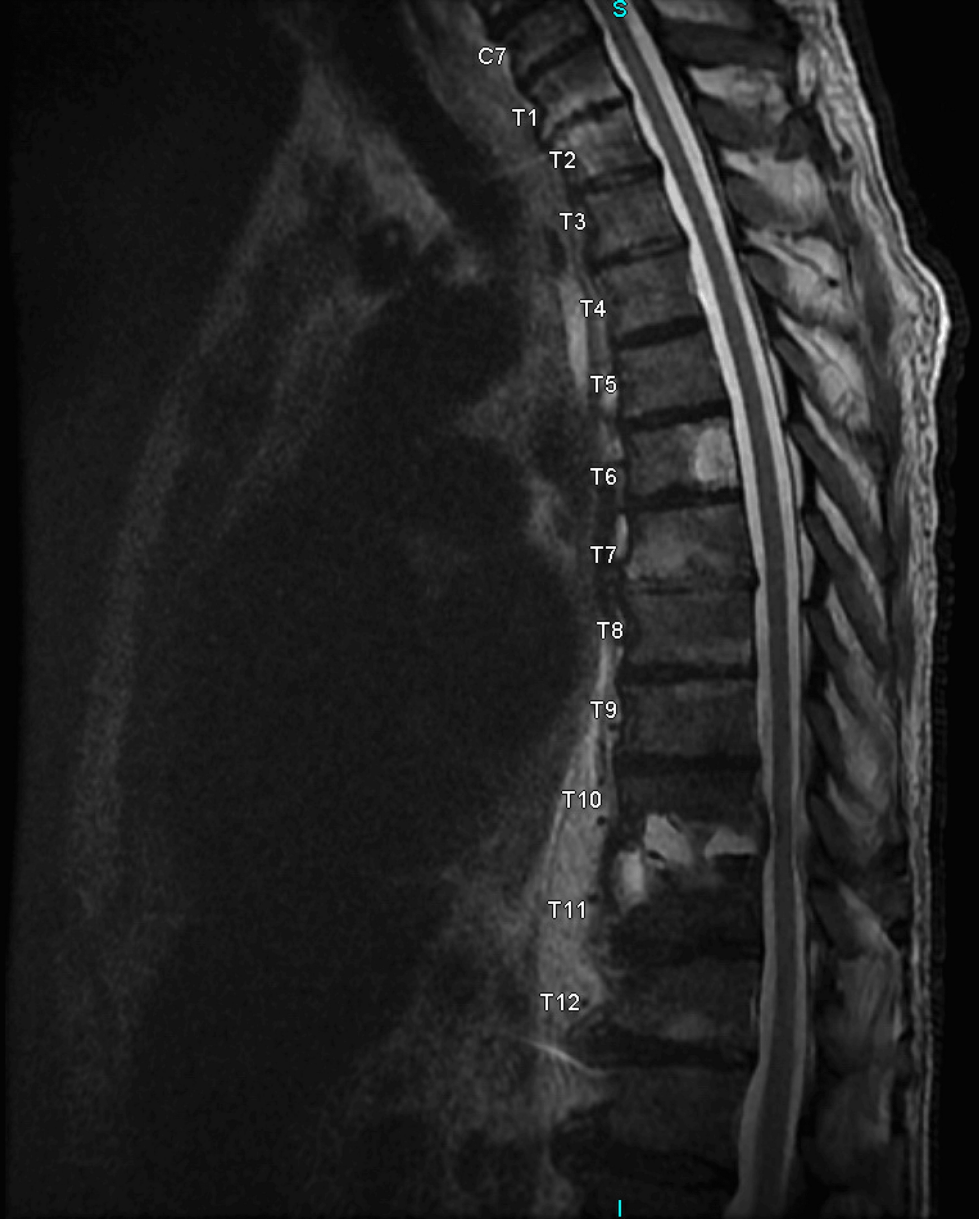
MRI of the thoracic spine without contrast Advanced vertebral body osteomyelitis and discitis at T10-11 with significant adjacent edema and inflammation. There is trace extension into the subligamentous spinal canal, including a posterior disc bulge and partial effacement of the thecal sac. A gadolinium enhancement is visible on T6 displaying a large hemangioma that is not clinically significant.

**Figure 4:**
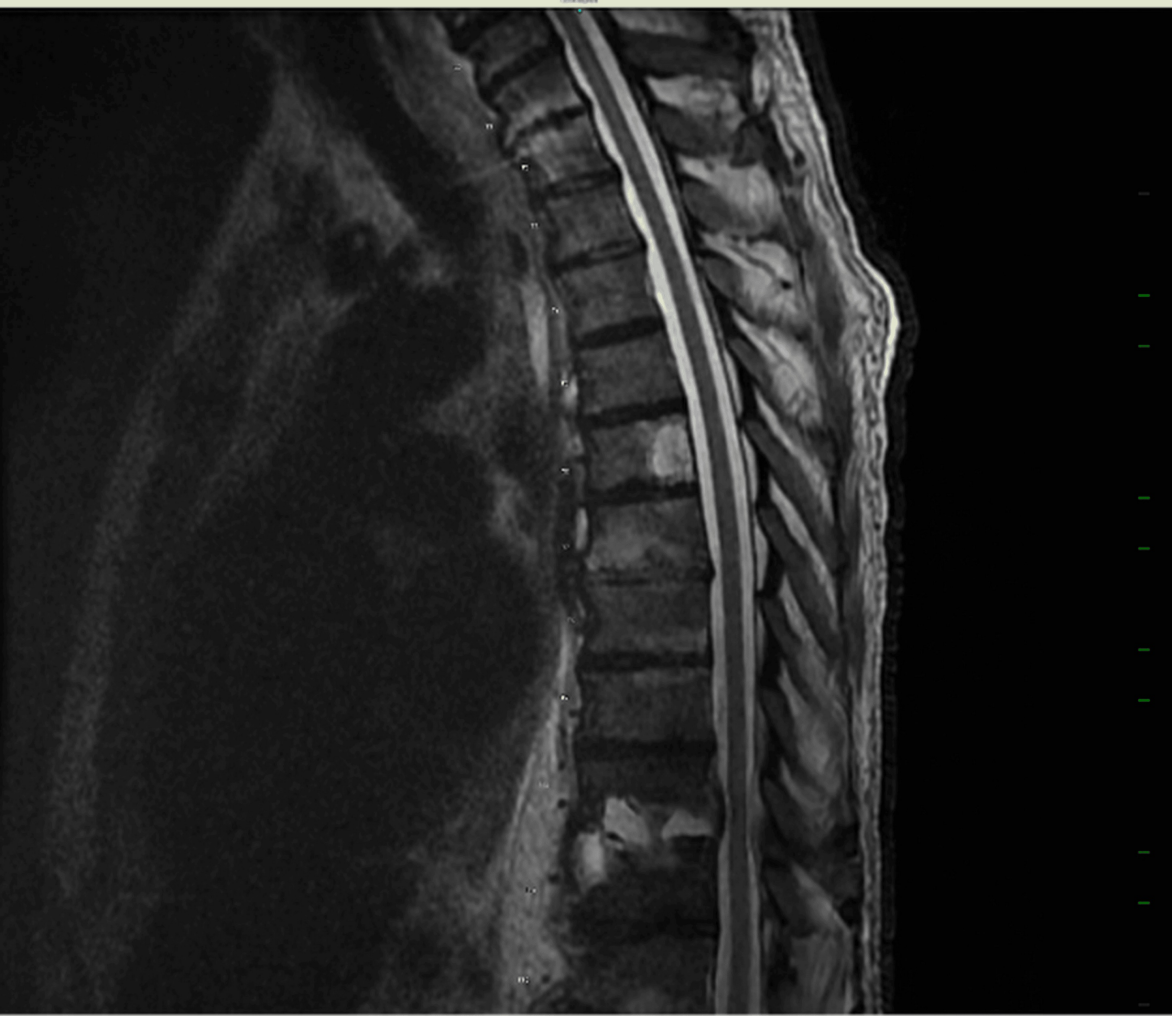
MRI of the thoracic spine without contrast Advanced discitis and osteomyelitis pattern at the T10-T11 level noted. There is suggestion of slight increase in epidural enhancement at the T10-T11 level with slight increased effacement of the thecal sac at this level. A gadolinium enhancement is visible on T6 displaying a large hemangioma that is not clinically significant.

On day 20 of the patient’s hospital stay, the patient’s neurological condition worsened, and he presented with the onset of bilateral lower limb flaccid paralysis, numbness, and weakness. An MRI of the thoracic spine with and without contrast revealed worsening osteomyelitis and discitis at T10-T11 (Figures 3, 4). Given these findings, neurology and orthopedic surgery were consulted. The neurology team diagnosed the patient with anterior cord syndrome. The orthopedic surgery team recommended decompression and a posterior laminectomy. Due to the patient’s pre-existing pulmonary issues, uncontrolled type II diabetes mellitus, and chronic kidney disease, the orthopedic surgery team decided that a transthoracic decompression would be potentially lethal in this case. Therefore they elected to proceed with a posterior decompression through the transpedicular route.

On day 21 of the patient’s hospital stay, the orthopedic surgery team performed a transpedicular spinal cord decompression, bilateral T10-T11 laminectomy, and partial T10 and T11 corpectomy. Next, an anterior column reconstruction with an antibiotic-impregnated calcium sulfate allograft material mixed with gentamicin and vancomycin was performed. An open biopsy of the T10 vertebral body was sent for histology and culture, which revealed the presence of MRSA (Table 2).

**Table 2:** T10 vertebral body tissue culture results with drug sensitivities MRSA: methicillin-resistant Staphylococcus aureus

Category	Details
Growth Indication	MRSA
Source	Surgical
Body Site	Thoracic Vertebrae
Specimen	T-10 Vertebral Body
Azithromycin	MIC Interpretation: R
Ceftaroline	MIC Interpretation: S
Chloramphenicol	MIC Interpretation: S
Ciprofloxacin	MIC Interpretation: R
Clindamycin	MIC Interpretation: S
Daptomycin	MIC Interpretation: S
Erythromycin	MIC Interpretation: R
Gentamicin	MIC Interpretation: S
Levofloxacin	MIC Interpretation: S
Linezolid	MIC Interpretation: S
Penicillin	MIC Interpretation: R
Rifampin	MIC Interpretation: S
Tetracycline	MIC Interpretation: S
Trimethoprim/Sulfamethoxazole	MIC Interpretation: S
Vancomycin	MIC Interpretation: S

Given the presence of bacteremia, a Hickman catheter was placed in the right subclavian vein for continued IV antibiotic treatment. On day 28 of the hospitalization, the patient was discharged to a long-term acute care (LTAC) facility for continued treatment.

## Discussion

The anterior spinal artery supplies blood to the anterior two-thirds of the spinal cord, which houses crucial neural circuits for function [3]. When the ASA is infarcted, the corticospinal tract is damaged, resulting in motor dysfunction beneath the lesion [3]. Additionally, osteomyelitis can disseminate into the epidural space, resulting in the formation of an abscess. It can also debilitate the vertebral bones, rendering them susceptible to collapse or instability, which can lead to misalignment and exert pressure on the ASA, disrupting the blood flow and causing ischemia, which in turn leads to motor dysfunction below the level of the lesion [3]. In addition to autonomic dysfunction (including orthostasis), bowel, bladder, and sexual dysfunction, loss of pain and temperature perception also result from damage to the lateral spinothalamic tract. However, vibration, proprioception, and two-point discrimination are maintained by the dorsal columns [3]. Imaging and clinical assessment are the main methods used to diagnose anterior cord syndrome [4]. MRI is the gold standard for identifying vertebral osteomyelitis because of its great sensitivity and potential to detect changes in soft tissue and bone structures early [4]. The patient’s MRI confirmed progressive osteomyelitis and discitis at the spinal level of T10 to T11.

Increased age, immunosuppression, uncontrolled diabetes, hypertension, long-term corticosteroid usage, malignancy, malnourishment, and intravenous drug use are risk factors for vertebral osteomyelitis [5]. The patient's advanced age, end-stage renal disease requiring dialysis, and type II diabetes mellitus, in addition to catheter manipulation, which may permit bacteria to enter the system and potentially lead to infection, probably contributed to the hematogenous spread of MRSA. Once infectious pathogens have access to the vertebral column, the infection can spread to adjacent paraspinal tissues, nerve roots, the epidural space, and even the intradural space; this creates inflammation, abscesses, and both soft tissue and osseous destruction [6]. This can subsequently lead to spinal cord infarction or compression by causing direct infection and inflammation that can compromise the spinal cord's blood supply [6]. The osteomyelitis infection caused compression and inflammation at the T10-T11 spinal level, which most likely caused the patient's paralysis and weakness.

The treatment of vertebral osteomyelitis requires intravenous antibiotic use for about four to six weeks, depending on the strain in question. In this instance, the treatment plan is guided by the results of the histology culture and the determination of susceptible antibiotics. Following this, a tailored combination of antibiotics can be formulated, as evidenced by the prescription of gentamicin and vancomycin in this particular case [6]. When conservative treatment fails or neurological deficits become apparent, spinal stabilization and decompression are required to reduce pressure on the spinal cord [7]. Due to deterioration in neurologic function, the patient underwent a posterior decompression procedure via the transpedicular route, which is minimally invasive and preserves spinal stability. This technique permits effective decompression of the spinal cord and nerves. This approach was done because of the patient’s pre-existing pulmonary issues, uncontrolled type II diabetes mellitus, and chronic kidney disease.

## Conclusions

Hospitalists, infectious disease specialists, neurosurgeons, and rehabilitation teams need to work together to facilitate early recognition of critical clinical symptoms in managing complex MRSA-induced spinal cord infections. The complexity of this case is due to the patient’s comorbid conditions.

Correlating the symptoms of constipation and bladder dysfunction to a neurogenic cause was a challenge in this patient in the setting of hypothyroidism and opioid usage. In summary, early detection of infection and promptly working with physical therapy are critical in order to prevent catastrophic neurological consequences in patients with multiple comorbidities.
